# Multiple non-ossifying fibromas as a cause of pathological femoral fracture in Jaffe-Campanacci syndrome

**DOI:** 10.1186/1471-2474-15-218

**Published:** 2014-06-26

**Authors:** Stéphane Cherix, Yann Bildé, Fabio Becce, Igor Letovanec, Hannes A Rüdiger

**Affiliations:** 1 Department of Orthopaedics and Traumatology, Lausanne University Hospital, Avenue Pierre-Decker 4, 1011 Lausanne, Switzerland; 2 Department of Diagnostic and Interventional Radiology, Lausanne University Hospital, Lausanne, Switzerland; 3 University Institute of Pathology, Lausanne University Hospital, Lausanne, Switzerland

**Keywords:** Jaffe-Campanacci syndrome, Multiple non-ossifying fibromas, Pathological fracture, Café-au-lait spots, Axillary freckles, Type 1 neurofibromatosis

## Abstract

**Background:**

Jaffe-Campanacci is a rare syndrome characterised by the association of café-au-lait spots, axillary freckles, multiple non-ossifying fibromas of the long bones and jaw, as well as some features of type 1 neurofibromatosis. There are less than 30 reported cases, and a genetic profile has not yet been determined. Furthermore, it has not been clarified whether it is a subtype of type 1 neurofibromatosis or a separate syndrome. The risk of pathological fracture is over 50%, due to substantial cortical thinning of the weight-bearing bones.

**Case presentation:**

A 17-year-old female patient, known for type 1 neurofibromatosis, presented with a low-energy distal femoral fracture due to disseminated large non-ossifying fibromas. Investigations revealed all of the distinctive signs of Jaffe-Campanacci syndrome. Both her distal femurs and proximal tibias exhibited multiple non-ossifying fibromas. The fracture was treated by open reduction and internal plate fixation. Some of the bony lesions were biopsied to confirm the diagnosis. The fracture healed eventless, as did the lesions biopsied or involved in the fracture. The other ones healed after curettage and bone grafting performed at the time of plate removal.

**Conclusion:**

Jaffe-Campanacci is a rare syndrome having unclear interactions with type 1 neurofibromatosis, which still needs to be characterised genetically. It is associated with a high risk of pathological fracture, due to the presence of multiple large non-ossifying fibromas of the long bones, with an expected normal healing time. Curettage and bone grafting promote healing of the lesions and should be considered to prevent pathological fracture. We agree with other authors that all patients with newly-diagnosed type 1 neurofibromatosis should undergo an osseous screening to detect disseminated non-ossifying fibromas, and evaluate the inherent risk of pathological fracture.

## Background

In 1958, Jaffe first reported the association of multiple non-ossifying fibromas (NOFs), café-au-lait spots and axillary freckles, suggesting an unusual form of type 1 neurofibromatosis (NF1) [1]. The definition “Jaffe-Campanacci syndrome” was proposed by Mirra et al. in 1982 [2]. In 1983, Campanacci et al. reported a series of ten patients meeting the criteria of this syndrome; it remains the largest series in the literature so far [3]. Only few other authors reported cases of this rare condition [4-9]. Colby et al. subsequently suggested the syndrome might be a distinct form of NF1 [10]. They assumed a genetic aetiology for this variability, and insisted on the necessity of a genetic analysis to confirm their hypothesis.

We report hereafter the case of a young patient presenting with a pathological femoral fracture as a consequence of Jaffe-Campanacci syndrome.

## Case presentation

**Figure 1:**
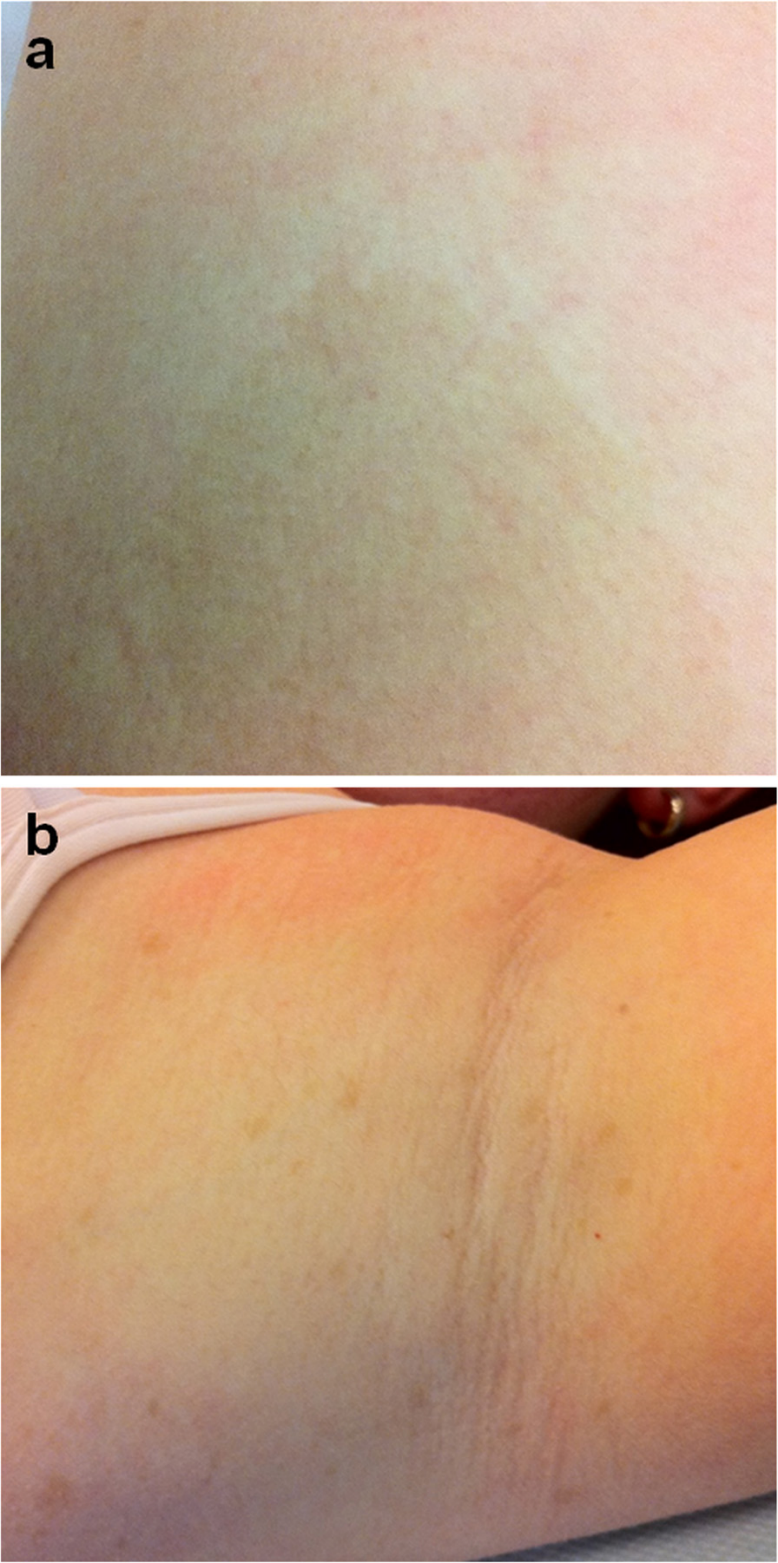
Typical “coast-of-California” café-au-lait spots (a) and axillary freckles (b).

**Figure 2:**
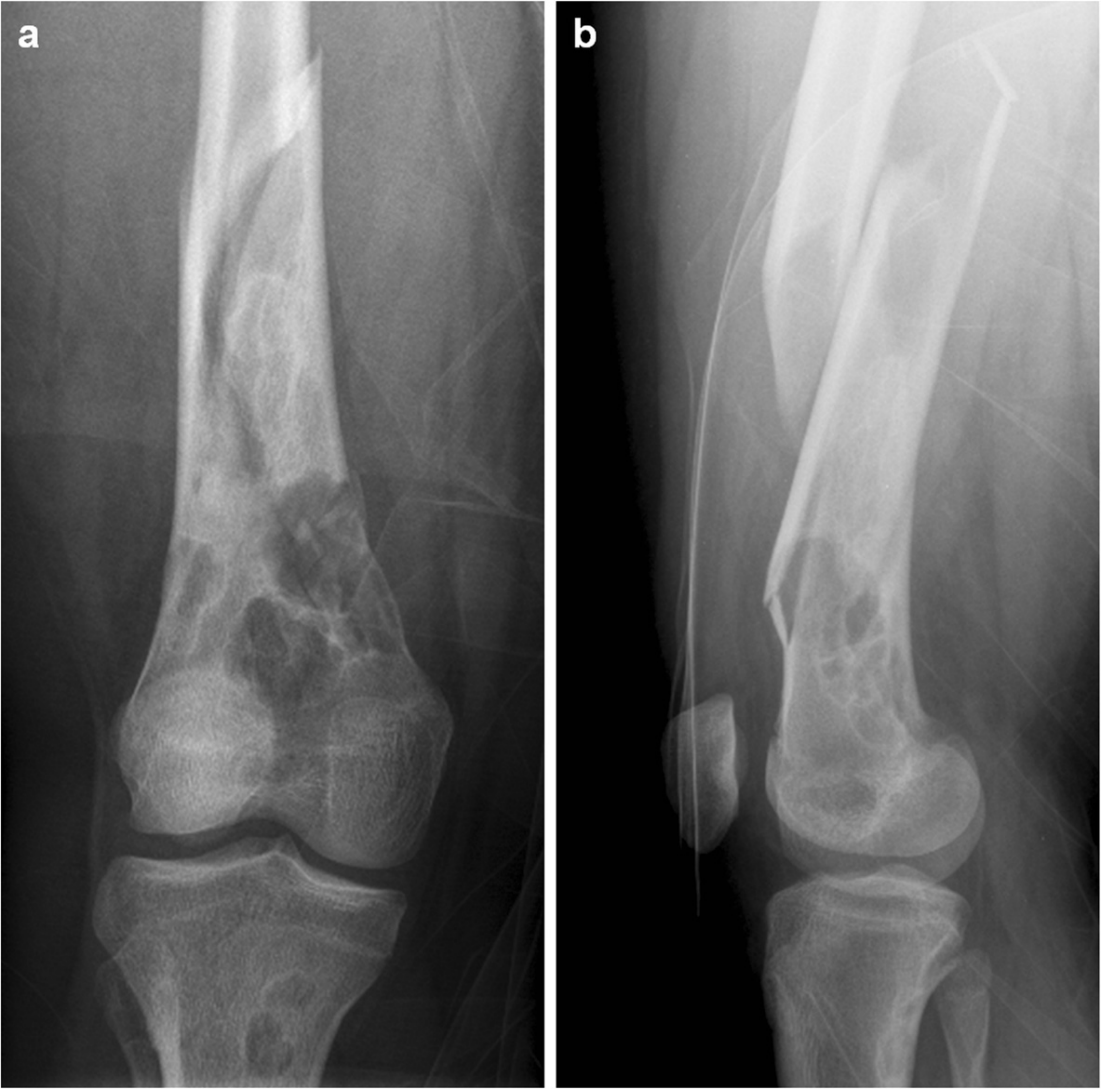
Anteroposterior (a) and lateral (b) radiographs displaying a pathological comminuted spiroid fracture of the right distal femoral diaphysis originating from a cluster of multiple large NOFs.

**Figure 3:**
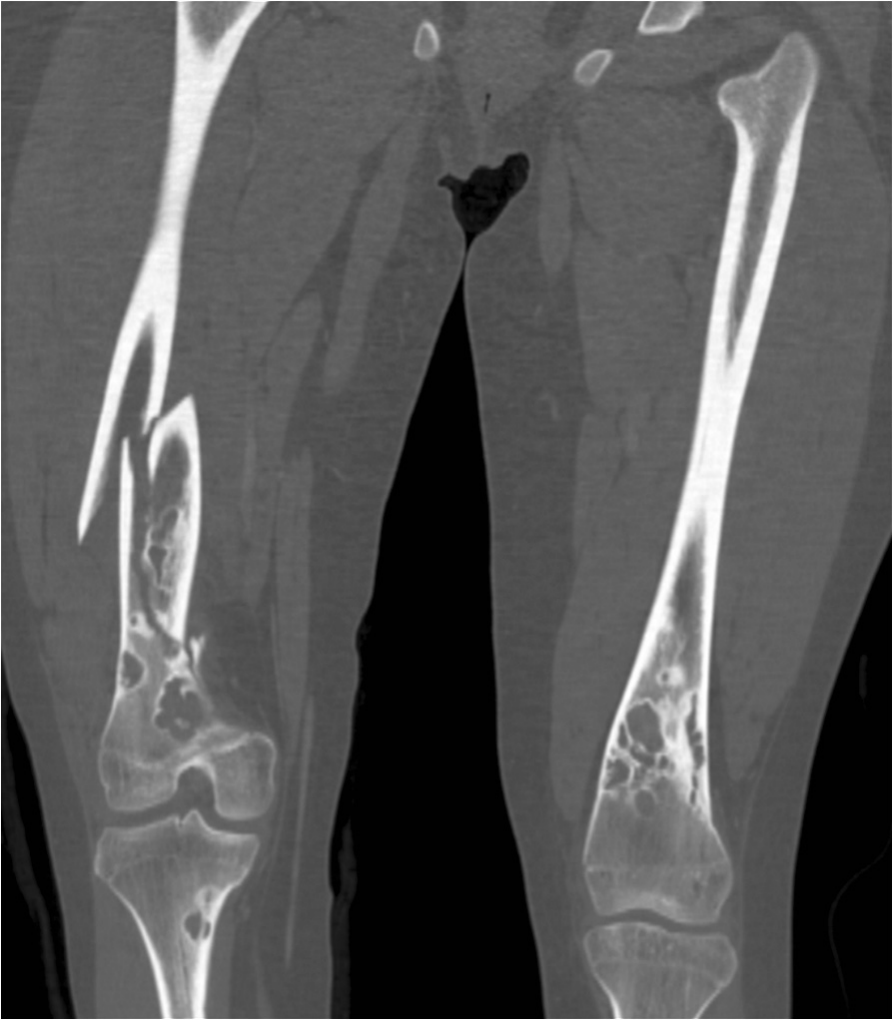
Coronal-reformatted CT image demonstrating multiple bilateral NOFs of both the distal femurs and proximal tibias.

**Figure 4:**
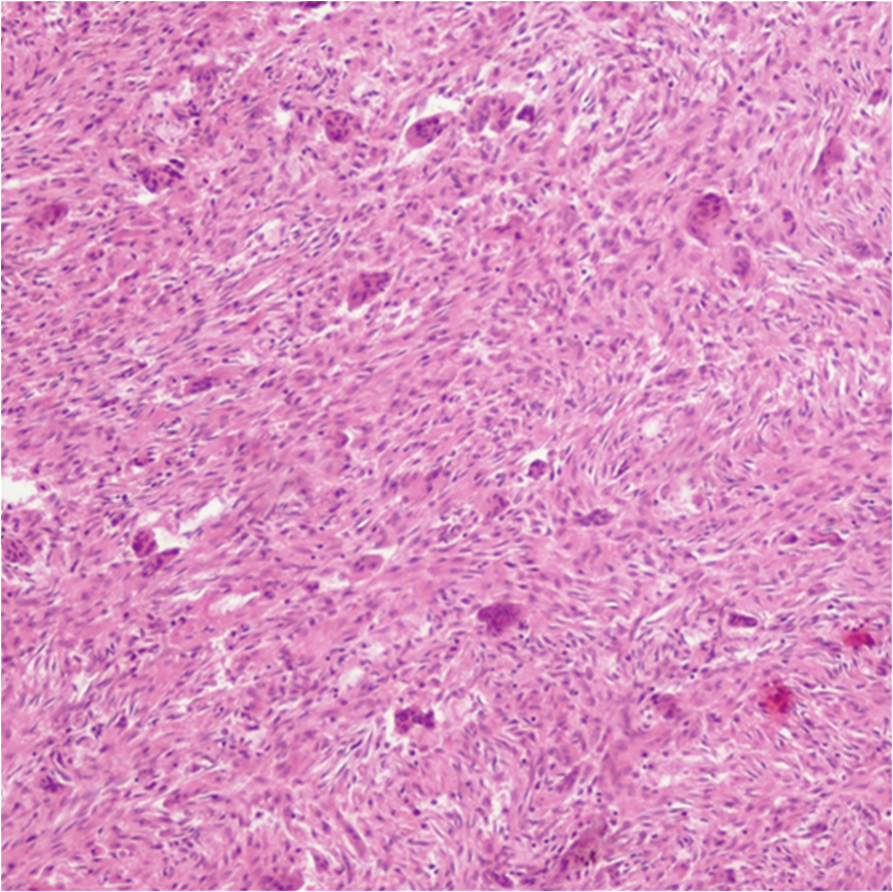
Biopsy specimen displaying spindle-shaped fibroblasts in storiform pattern and multinucleated giant cells, typical for NOF.

**Figure 5:**
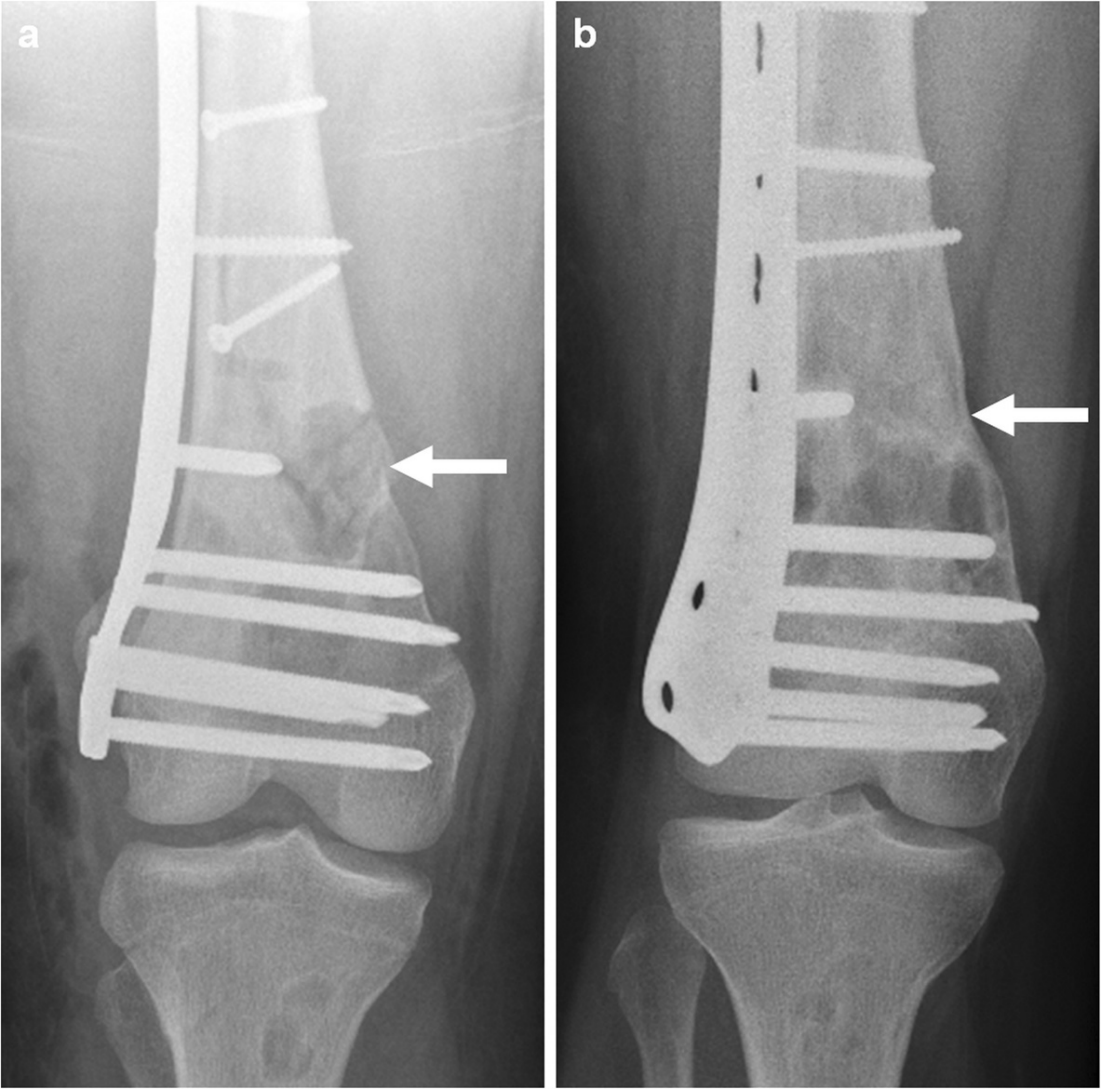
Post-operative (a) and 18-month follow-up (b) radiographs illustrating the complete healing of one biopsied NOF (arrow), whereas the others remain unchanged.

**Figure 6:**
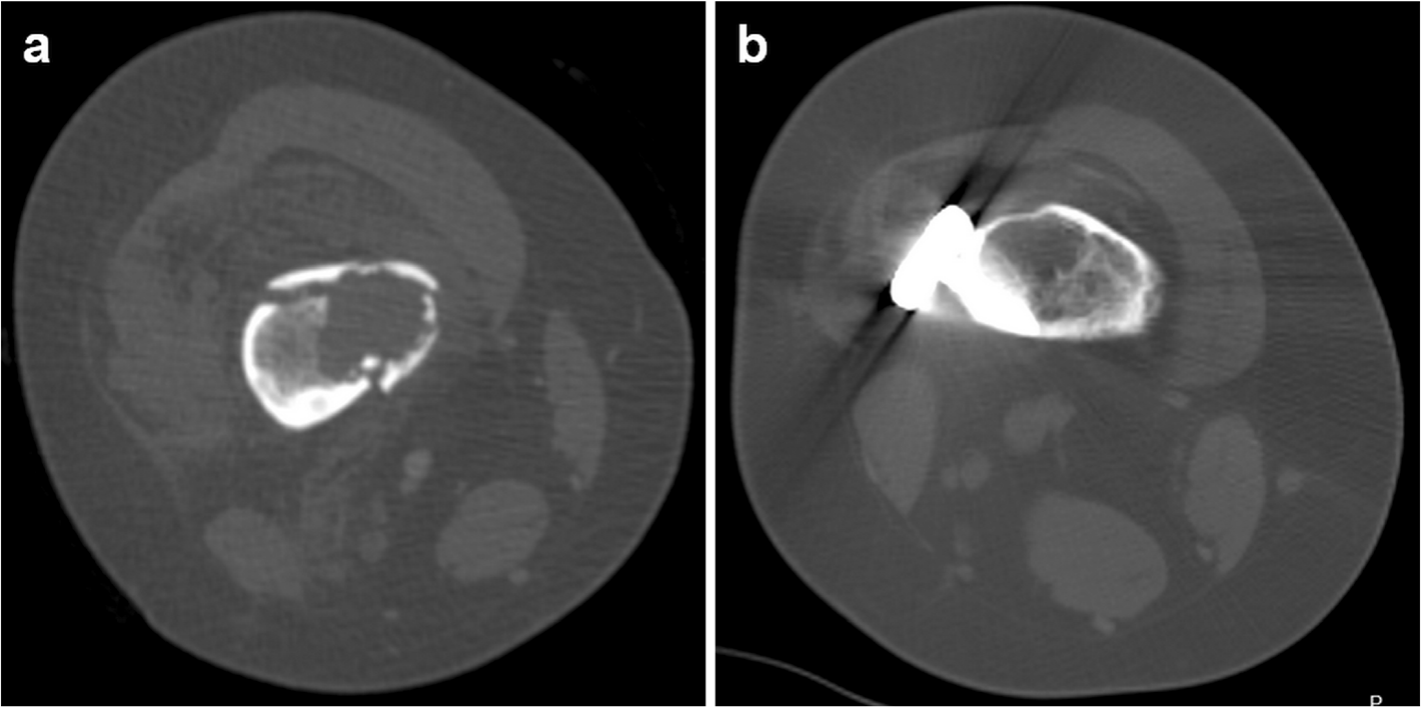
Pre-operative (a) and 18-month follow-up CT (b) demonstrating the healing of one biopsied NOF.

**Figure 7:**
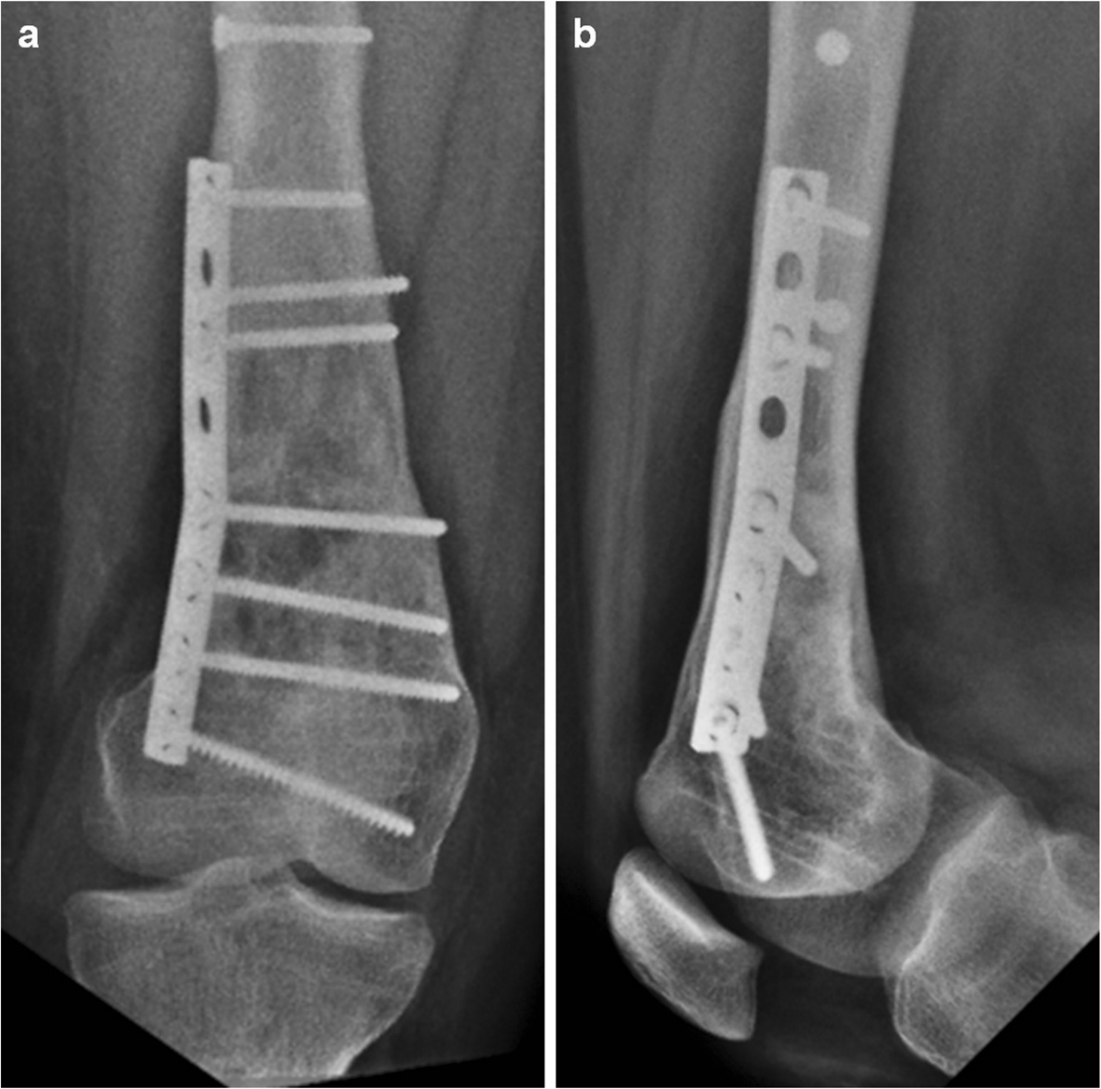
Anteroposterior (a) and lateral (b) radiographs performed 6 months after plate removal, curettage, bone grafting of the remaining NOFs and prophylactic fixation of the distal femur.

A 17-year-old female patient, suffering from NF1, sustained a fracture of her right femur due to a low-energy accident while skiing. Skin examination revealed multiple “coast-of-California” café-au-lait spots on the abdomen, the back and all four limbs (Figure 1). There were also bilateral axillary freckles; interestingly, no neurofibroma was observed. Medical history included low height and mild mental retardation. An ophthalmological examination revealed a Lisch nodule. A trans-oesophageal echocardiography showed no cardiac abnormality. Family history revealed that her father suffered from NF1. He had not been treated for any fracture or musculoskeletal disorder.Conventional radiographs displayed a comminuted spiroid fracture of the right distal femoral diaphysis, originating from a cluster of osteolytic lesions of the metaphyseal region (Figure 2). These lesions were rounded, of variable size, well defined, mostly with sclerotic margins, corresponding to typical NOFs. The fracture originated from a large lesion that had produced scalloping and weakening of the medial femoral cortex. Computed tomography (CT) scan of the entire skeleton revealed that both her distal femurs and proximal tibias presented multiple NOFs (Figure 3). There were no other abnormalities on thoraco-abdominal CT.The fracture was treated by open reduction and internal plate fixation. Biopsy, harvested at the time of osteosynthesis, confirmed the diagnosis of NOF (Figure 4). The fracture healed without complications, and the patient returned to work within 4 months. Post-operative radiographs at 18 months revealed complete consolidation of the fracture and ossification of some of the NOFs (Figures 5 and 6). The others healed after curettage and bone grafting performed at the time of plate removal (Figure 7).

## Discussion

It remains intriguing whether the Jaffe-Campanacci syndrome is a particular form of NF1 or a separate entity. In the 2013 “WHO Classification of tumours of soft tissue and bone”, it is defined as the association of NOFs and NF1 [11]. Colby et al. suggested the syndrome might be a manifestation of the variability of NF1, as all four patients of their series met the criteria of both diseases [10]. This clinical overlap brought them to suggest allelic proximity. Unfortunately, a genetic analysis was not performed, and hence this hypothesis has never been confirmed.

While in our patient the presence of NF1 was genetically confirmed, neither skin examination nor a whole-body CT scan revealed any evidence of neurofibromas. However, the patient met all the criteria for Jaffe-Campanacci syndrome, as proposed by Mirra et al. [2], including axillary freckles, café-au-lait spots and multiple NOFs. We agree with Colby et al. that genetic assessment is critical to better define this syndrome [10].

The exact prevalence of NOFs is unknown, as the vast majority remains undiagnosed; the estimated rate is 30-40% of the children [12,13]. Multiple NOFs are far less common, and their association with NF1 is extremely rare; in a series of 900 patients with biopsy-proven NOFs, Moser et al. found 72 cases (8%) with multiple lesions, from which 4 (0.4%) were associated with NF1 [14]. In their retrospective review of 401 patients with bone lesions characterised as NOFs or similar lesions, Mankin et al. found only 2 cases (0.5%) meeting the criteria for Jaffe-Campanacci syndrome [8].

The fracture risk appears to be high in Jaffe-Campanacci syndrome, since more than half of the patients will experience at least one fracture (7/10 patients in the series of Campanacci et al. [3], and 6/14 cases reported by other authors [4-10]). Colby et al. reported on two femoral fractures in four patients; they suggested the higher fracture risk may be due to multiple large lesions, with thinning and weakening of the cortices of the weight-bearing bones, mainly the proximal or distal femur and the proximal tibia [10]. They proposed to screen NF1 patients for NOFs, to detect those at higher risk of pathological fracture and discuss prophylactic fixation.

In most reported cases of pathological fractures associated with Jaffe-Campanacci syndrome, internal fixation was necessary [3,10]. In our patient, the fracture necessitated open reduction and internal plate fixation. We performed a biopsy of the more easily accessible lesions to confirm the diagnosis. The NOFs involved in the fracture, or which were biopsied, almost completely ossified with time. In contrast, the lesions that were not biopsied remained mostly unchanged. They healed after curettage and bone grafting at the time of plate removal.

Cases of limb deformities due to NOFs have been described [15]. Even in the absence of a relevant fracture risk, surgical treatment might be advised to avoid progression of deformities.

## Conclusions

Jaffe-Campanacci is an ill-defined syndrome associating multiple NOFs, skin manifestations (café-au-lait spots and axillary freckles) and some features of NF1. NOFs are typically large and located on the distal femur and/or proximal tibia. Due to the high incidence of pathological fractures, this risk should be regularly evaluated and the affected bone stabilised if necessary [16]. During internal fixation, biopsy or curettage and bone grafting should be systematically considered to promote healing.

## Consent

Written informed consent was obtained from the patient (aged 19 years at the time of signature) for publication of this Case report and any accompanying images. A copy of the written consent is available for review by the Editor of this journal.

## Abbreviations

CT: Computed tomography; NF1: Type 1 neurofibromatosis; NOF: Non-ossifying fibroma; WHO: World Health Organization.

## Competing interests

The authors declare that they have no competing interests.

## Authors’ contributions

SC treated the patient, took the clinical pictures of the patient, and wrote the manuscript. YB made the literature review and revised the manuscript. FB prepared the medical imaging section including radiological images, and revised the manuscript. IL produced the histopathological diagnosis and images, and revised the manuscript. HAR leads the oncologic orthopaedic section of our institution and revised the manuscript. All authors read and approved the final manuscript.

## Pre-publication history

The pre-publication history for this paper can be accessed here:

http://www.biomedcentral.com/1471-2474/15/218/prepub
